# Thromboelastographic analysis of novel polyethylene glycol based low volume resuscitation solutions

**DOI:** 10.1371/journal.pone.0207147

**Published:** 2018-11-15

**Authors:** Loren K. Liebrecht, Jason Newton, Erika J. Martin, Nina Wickramaratne, Sudha Jayaraman, Jinfeng Han, Michel Aboutanos, Donald F. Brophy, Martin J. Mangino

**Affiliations:** 1 Department of Surgery, Division of Acute Care Surgery, Virginia Commonwealth University School of Medicine, Richmond, Virginia, United States of America; 2 Department of Biochemistry and Molecular Biology, Virginia Commonwealth University School of Medicine, Richmond, Virginia, United States of America; 3 Department of Pharmacotherapy and Outcomes Science, Virginia Commonwealth University School of Pharmacy, Richmond, Virginia, United States of America; 4 Department of Physiology and Biophysics, Virginia Commonwealth University School of Medicine, Richmond, Virginia, United States of America; 5 Department of Emergency Medicine, Virginia Commonwealth University School of Medicine, Richmond, Virginia, United States of America; University of South Carolina, UNITED STATES

## Abstract

**Background:**

Low volume resuscitation (LVR) in shock prevents deleterious effects of crystalloid loading in pre-hospital settings. Polyethylene glycol 20,000 (PEG-20k) based LVR solutions are 20-fold more effective at maintaining perfusion and survival in shock compared to conventional crystalloids. The aim of this study was to determine coagulation and platelet function of whole blood treated with 10% PEG-20k.

**Methods:**

Citrated blood from volunteers (n = 25) or early admission severely injured trauma patients (n = 9) were diluted 10% with various LVR solutions in a matched design with a paired volume control (saline), and studied using thromboelastography (TEG).

**Findings:**

In healthy volunteers and patients, 10% PEG-20k significantly increased clot amplification time (k), decreased propagation (angle), maximal clot size and strength (MA), and the overall coagulation index (CI), but not clot initiation (R) or fibrinolysis (Ly30), relative to paired saline dilutional controls. Clinically, K, angle, and MA were just outside of the normal limits in volunteers but not in patients. No statistical differences existed between PEG-20k and Hextend (HES) in either patient population. In a dose response series using volunteer blood, all effects of 10% PEG-20k on TEG were reversed and normalized by lower concentrations (7.5% and 5%). Furthermore, 7.5% PEG-20k produced similar resuscitation effects as 10% PEG in rodent hemorrhagic shock models (n = 5).

**Conclusions:**

In conclusion, PEG-20k based LVR solutions produced a dose-dependent minor hypocoagulative state, possibly associated with changes in clot propagation and platelet function, which can be reversed by dose reduction in concentration while providing superior LVR, microvascular rescue, and lactate clearance compared to saline or starch.

## Introduction

Minimizing the use of crystalloids and utilizing blood products after trauma are now becoming mainstream in civilian trauma centers. Damage control resuscitation is also emerging as standard of care for the US Department of Defense, according to the Joint Theater Trauma Systems Clinical Practice Guidelines (JTTS CPG). When blood products are not available for resuscitation, crystalloid solutions are administered. However, only a fraction of infused conventional crystalloid volume stays in the intravascular space so the use of low volume crystalloids has minimal effects on pressure and perfusion [1, 2]. The movement of crystalloid fluid from capillary to interstitium is compounded by the increase in capillary permeability from trauma-related inflammation and trauma-induced capillary leak syndrome (TICS) [3]. Furthermore, crystalloid resuscitation exacerbates TICS, acidosis, hypothermia, and coagulopathy [3, 4]. Other resuscitation solutions such as hypertonic saline or starch have had disappointing results [5, 6] including concerns and risks associated with their use [4, 7]. There remains a need for a better crystalloid fluid that can be given at a low volume to resuscitate patients in severe hemorrhagic shock awaiting definitive treatment, especially for the prehospital setting.

Recently, polyethylene glycol (PEG) polymers of specific molecular weight ranges have been used in crystalloid solutions to act as highly effective Low volume Resuscitation (LVR) solutions [2, 8–10]. These polymers non-energetically move isotonic fluid from intracellular and interstitial spaces into the capillary space by osmotic flow mechanics in response to metabolic cell swelling that occurs in shocked and ischemic tissues. During low-oxygen states, energy-dependent ion homeostasis break down and membrane function becomes dysregulated, allowing an imbalance of solute and solvent within these spaces. Cellular swelling, apoptosis, necrosis, and a loss of capillary flow dynamics perpetuates the cycle of shock [4, 7]. Reversing and preventing these effects may be possible with protective resuscitation solutions containing polyethylene glycol (PEG) that alters fluid volume transfer in cell and extracellular spaces during shock.

PEG-20k molecules are impermeant to cells and selectively partition in the capillary and interstitial spaces in a ratio of 2:1, respectively [2, 10]. This pulls isotonic fluid out of swollen cells and tissues. As water flow moves from the interstitial spaces to the capillaries, the capillary exchange in the tissues dramatically improves under very low volume conditions because the microcirculation is decompressed, lowering the resistance to flow, while the capillary spaces are re-loaded with volume and pressure for driving flow [10]. This causes rapid clearance of lactate, increased blood pressure, and tolerance to the low volume state [8]. Thus, despite being in a post-hemorrhagic state under very low volume conditions with dysregulated membrane mechanisms, polyethylene glycol was able to decompress the microcirculation to improve capillary exchange in the tissues and dramatically optimize resuscitation. Interestingly, the polyethylene glycol polymers were shown to work several fold better than hydroxyethyl starch based solutions in these hemorrhagic resuscitation studies, implying different mechanisms of action of the polymers, despite theoretically similar mechanisms of exerting oncotic pressures intravascularly. Differences in underlying biochemical properties, size, and structure are likely to play a large role in these differences by causing them to partition differently in the microcirculation and pull water at different rates. Additional differences may also include vascular interactions with the glycocalyx and capillary structures in varying shock and ischemic states or the formation of hydration shells [11, 12].

Intravenous administration of Hextend (hydroxyethyl starches) have well known complications of both renal toxicity and coagulopathies [13], which in trauma or acute care settings are definite concerns, especially given the expanding literature on trauma related coagulopathy or shock induced endotheliopathy [13]. Interference with blood clotting and coagulation may be shared by both PEG and starch polymers, due to hemodilution or other cell-mediated causes, despite the aforementioned differences. Therefore, the purpose of this study was to examine any possible effects of PEG-20k based LVR solutions on overall whole blood coagulation and platelet function in both healthy volunteers and a selection of trauma patients using thromboelastography.

## Methods

### Low volume resuscitation (LVR) solutions

Normal saline (0.9% NaCl, NS; prepared using 9 g/L sodium chloride) served as both a crystalloid vehicle volume control (as all solutions were prepared in a normal saline base), and as a dilutional volume control (as all blood samples were diluted 10% with each solution, to represent the 10% volume administered after hemorrhagic shock in all previous rodent and porcine LVR studies) [1, 2]. A military medicine resuscitation comparative control used a 6% hydroxyethyl starch (HES) solution and was purchased from the manufacturer under product name HEXTEND (HS), formulated with 6% hetastarch [molecular weight (MW) ~600 kDa (range 450–800 kDa) with ~0.75 molar substitution at primarily the C-2 glucose unit] in 0.9% sodium chloride. The experimental solutions consisted of polyethylene glycol (PEG) 20,000 mw (PEG-20k) dissolved in normal saline at concentrations of 10%, 7.5%, and 5%. Polyethylene Glycol-20k was purchased from Sigma Chemical Co (St. Louis, MO) as the molecular biology grade material. All solutions were either prepared fresh or filter sterilized using 0.22 micron filtration for storage in polypropylene containers to exclude bacterial or polymer degradation.

### Preparation of blood and TEG assay

An internally matched comparative analysis was designed where each study participant would serve as their own control. Each enrolled study participant’s blood was diluted 10% with each studied resuscitation fluid (NS, HS, PEG), always including a saline (NS) dilution control paired with 6% HES, and 10% PEG-20k. A dose-response series was also conducted using PEG-20k at concentrations of 5%, 7.5%, and 10% in saline at the same 10% dilution with whole blood. Both healthy volunteers and trauma patients were enrolled and consented under an approved Virginia Commonwealth University (VCU) IRB protocol. Healthy volunteers were without comorbidities or any medications and between 18–50 years of age. Trauma patients were those of the same age arriving at the highest alert level to our trauma system with any injury or mechanism, as long as they had evidence of severe hypovolemia or ischemia represented by a systolic blood pressure below 95 mm Hg, a plasma lactate level ≥ 4.6 mM, or an injury severity score (ISS) greater than 24. Blunt and penetrating trauma were included. Trauma patient blood was collected early after arrival, usually within 30 minutes and prior to any blood transfusions or significant crystalloid infusions. The goal was to select only those patients that received only small amounts (0–300 ml) of saline or Lactated Ringer’s (LR) crystalloid in the field or en-route to the emergency department. Patients who received larger fluid volumes or blood products were excluded from the study.

Venous blood samples from individual healthy volunteers or from trauma patients were drawn into citrate treated vacutainer tubes (15 ml total), pooled, and diluted 10% with saline, 6% Hextend, 10% PEG-20k, 7.5% PEG-20k, or 5% PEG-20k. Prior written consent was obtained from the volunteers and after the fact written consent was obtained from the trauma patients or their legal advocate. In cases where consent was either denied or not obtained after the fact from the patients, the blood samples and any results obtained from them were destroyed. Some TEGs were run on undiluted whole blood. They were then gently mixed by inversion, and analyzed on a TEG-5000 thromboelastograph (Haemonetics Corp.) within 2 hours from blood draw using kaolin activation by trained laboratory staff on machines calibrated daily. Each blood sample was analyzed by TEG twice and the values averaged. Each unique patient or volunteer blood sample was the source for all dilutions and the experimental values were compared to the same blood either undiluted or diluted equally with the vehicle (saline). Therefore, each sample served as its own control, which would take into account any variations that might be present in the blood sample hematocrits, platelet counts, or plasma protein samples (such as fibrinogen). The TEG data were reported as six outcome parameters that describe different functional attributes of the clotting and coagulation system of whole blood under these conditions. These include: **R**, a measure of the time to initiate fibrin clot formation; **k**, time to achieve a predetermined clot size and strength (20 mm clot size). This represents amplification of the clotting cascade; **Alpha (α)** or angle of the slope between R and k, which characterizes the propagation phase and thrombin burst converting fibrinogen into fibrin with fibrin cross linking; **MA**, the maximum amplitude of the clot that represents clot strength, which is generally composed of 80% platelet and 20% fibrin responses; **LY30**, the % lysis of the clot 30 min after maximal formation (MA), which represents rates of fibrinolysis of the clot; and **CI**, the coagulation index that is a mathematical model of overall coagulation responses using the other TEG parameters.

### Shock resuscitation testing

To test resuscitation outcomes of LVR solutions specifically for comparison with TEG outcomes in the dose response series of experiments, a standard rat lactate controlled model of severe hemorrhagic shock with low volume resuscitation was used as previously described in great detail elsewhere [2, 9, 10]. These studies were approved by the VCU IACUC and followed the ARRIVE guidelines [14]. Briefly, we determined tolerance to the low volume state in severely shocked acutely anesthetized rats (n = 5 for each group). Arterial bleeding to a mean arterial pressure of 35 mmHg was maintained until plasma lactate rose to 9–10 mM, which initiated low volume resuscitation using saline control, or 10% and 7.5% PEG-20k solution, all given intravenously at a volume equal to 10% of the estimated blood volume of the rat [15]. Immediately after LVR solutions are given, lactate falls but then begins to rise again until it again reaches the 9–10 mM limit. The time from the start of LVR infusion until the lactate rises back to its limit again (9–10 mM) is recorded as the LVR time. The LVR time and the lactate and MAP values at the end of the LVR time are all outcome measures of the tolerance to the hypotensive state.

### Statistical analysis

All statistical analyses were performing using GraphPad Prism version 6.07 for Windows (GraphPad Software, La Jolla California USA, www.graphpad.com). Data groups were analyzed for outliers using the nonlinear regression ROUT method with Q = 1%, the maximum desired false discovery rate. Normality of Gaussian distribution was then assessed using the D’Agnostino-Pearson ombinus K2 method. Most data were then analyzed by the non-parametric ANOVA Kruskal-Wallis test with the Mann-Whitney U test for multiple comparisons of means. Most TEG data are expressed as the median with the 2^nd^ and 3^rd^ interquartile ranges and the upper and lower extremes (box and whiskers). Significant differences from clinical limits of normal for laboratory values were determined using the Wilcoxon Signed Rank Test. Population data is expressed in mean ± SD.

## Results

### Solutions

All solutions were prepared with a base of NaCl that gave a final concentration of Na = 154 mEq and Cl– 154 mEq. The final solution calculated osmolarity of the three solutions that were used in most of these studies were; Saline vehicle control = 308 mOsm, 6% Hextend = 308.1 mOsm, and 10% polyethylene glycol 20k = 308.5 mOsm. The calculated osmotic pressure of each component was 15.06 Atm for the NaCl vehicle component, 0.122 Atm for the PEG-20k component in the PEG-20k solution, and 0.0027 Atm for the hydroxyethyl starch component of the Hextend solution (assuming an average molecular weight of the HES of 550,000 Da).

### Populations

The healthy volunteer population (n = 25) was enrolled intermittently between 7/2015-7/2017. Ages ranged 20–45 (28.4 ± 6.21), and 14/25 (56%) were males. Of note, HES colloidal comparative controls had n = 7 sample. The dose response group for PEG concentrations had n = 9 sample. All PEG or HS samples were matched with saline control. The trauma patient population (n = 9) was enrolled intermittently between 10/2016 and 5/2017. Ages ranged 18–39 (29 ± 8.8) years, and 7/9 were males. Penetrating injuries with or without polytrauma were seen in 3/9 patients (due to gunshot wounds to the trunk), while 6/9 patients presented with blunt/polytrauma injuries (due to motor vehicle or motorcycle collisions, or a 40ft fall in one case). Injuries were widespread including visceral lacerations (5/9), orthopedic fractures (5/9), hemo/pneumothorax or pulmonary contusions (5/9), burn (1/9), and traumatic brain injuries (4/9). Lowest pre-hospital or ER systolic blood pressure (SBP) ranged 50–124 (90.8 ± 1.56, n = 9) mmHg, while diastolic BP ranged 24–82 (60.8 ± 1.56, n = 9). Plasma lactate ranged 1.1–5.3 (3.3 ± 1.56, n = 5) mmol/L. ISS ranged 9–48 (30.2 ± 13.6, n = 9). The main purpose of the trauma patient group was to examine how their blood responded to PEG-20k LVR solutions and not necessarily to directly compare them to the volunteers or to show a trauma-induced coagulopathy.

### Initiation of clotting

**Fig 1:**
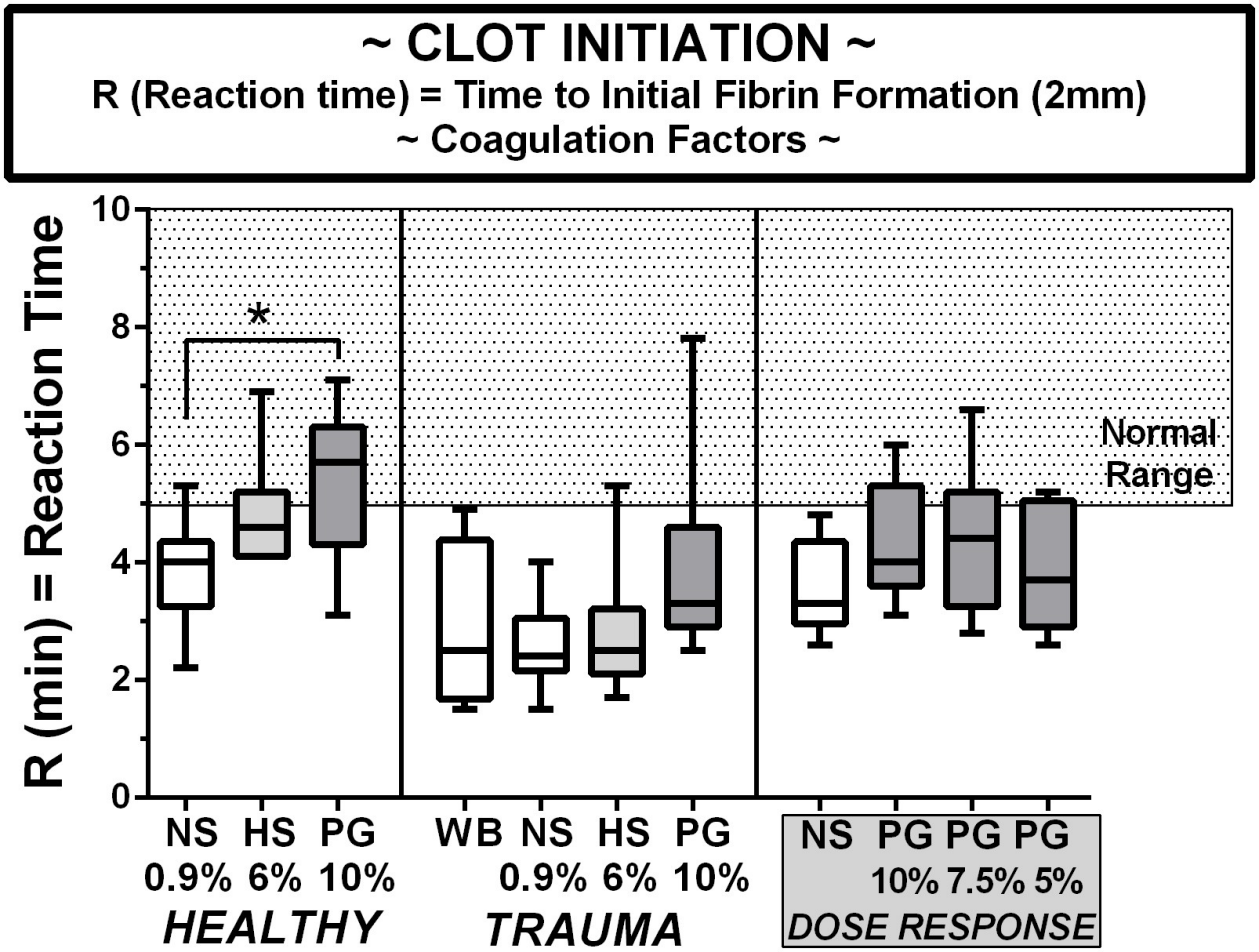
Clot initiation indexed by the R time on TEG in blood from healthy volunteers, trauma patients, and volunteers in a PEG-20k dose-response series. All TEG outcomes represents 25 healthy volunteers, 9 trauma patients, and 9 volunteers for dose-response studies. Whole citrate preserved blood was immediately diluted 10% with a saline vehicle (NS), 6% solution of Hextend (HS), and Polyethylene Glycol-20k (PEG) at concentrations of 10%, 7.5%, or 5% and assayed by full thromboelastography (TEG) within 2–3 hours of blood draw in a matched design with saline always serving as control to the resuscitative fluid. All values are expressed in a box and whiskers standard format where the bar in the box is the sample median value, the lower border of the box is the value demarcating the 1^st^ and second interquartile range, the upper border of the box is the value demarcating the 3^rd^ and 4^th^ interquartile range, and the upper and lower whiskers are the samples highest and lowest values, respectively. The shaded box represents the known normal ranges for the TEG values. WB = whole blood (undiluted). *P<0.05.

The TEG R time is shown in Fig 1. For healthy volunteers using 10% diluted whole blood, mean initiation times were lower than the normal range for both saline and HES diluted whole blood, significantly so for the saline, while being just within normal limits in PEG diluted blood. PEG significantly lengthened the initiation time, relative to the saline dilutional controls. Similar trends were seen in both diluted and whole blood from trauma patients, where all of the R times were below normal. For PEG dose responses, all values were below normal irrespective of PEG concentration ranges from 5–10%. The volunteers and patients showed similar R times for all groups. All normal TEG values were reported from Haemoinetics, Inc.

### Amplification of clotting

**Fig 2:**
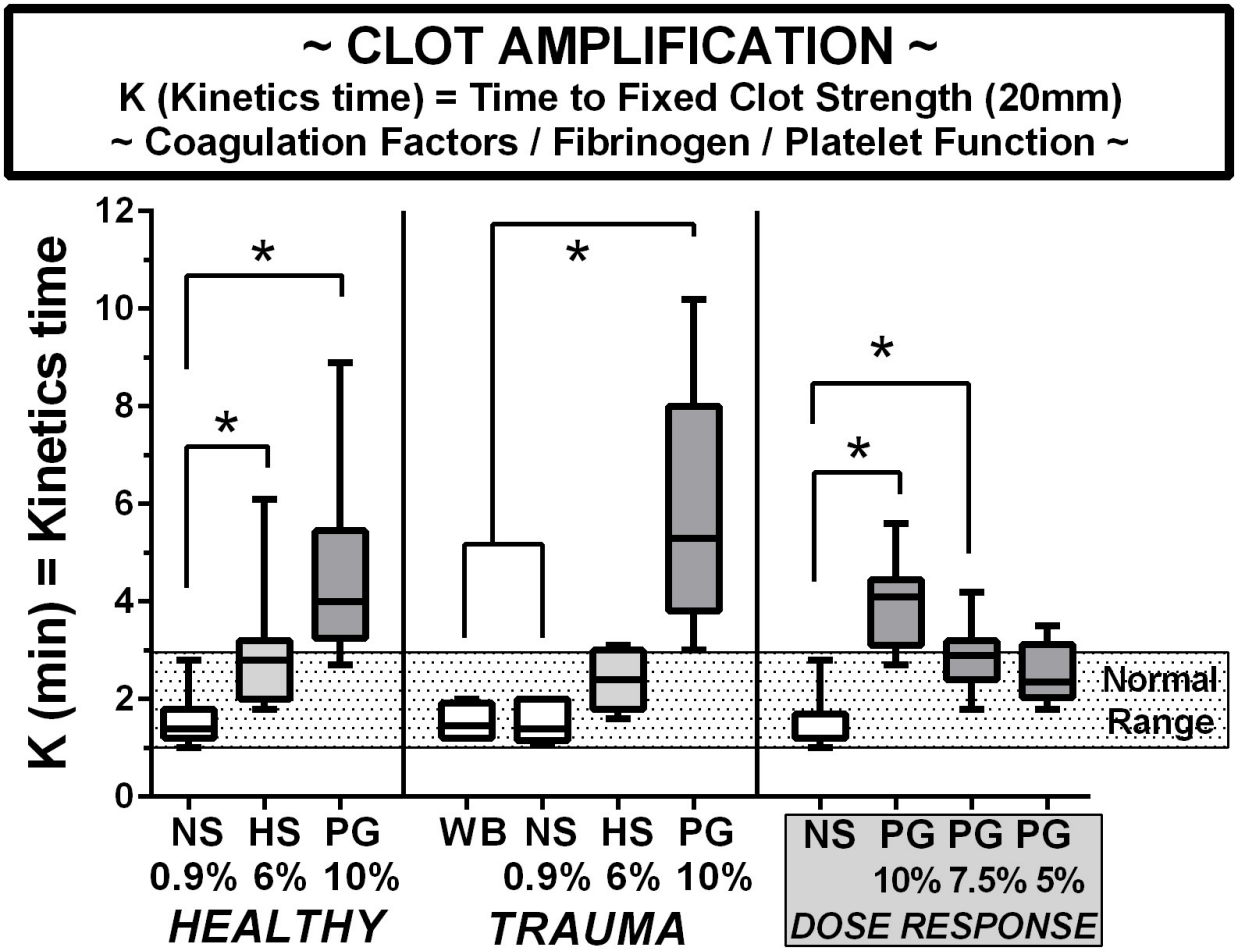
Clot amplification indexed by the k time on TEG in blood from healthy volunteers, trauma patients, and volunteers in a PEG-20k dose-response series. All TEG outcomes represents 25 healthy volunteers, 9 trauma patients, and 9 volunteers for dose-response studies. Whole citrate preserved blood was immediately diluted 10% with a saline vehicle (NS), 6% solution of Hextend (HS), and Polyethylene Glycol-20k (PG) at concentrations of 10%, 7.5%, or 5% and assayed by full thromboelastography (TEG) within 2–3 hours of blood draw in a matched design with saline always serving as control to the resuscitative fluid. All values are expressed in a box and whiskers standard format where the bar in the box is the sample median value, the lower border of the box is the value demarcating the 1^st^ and second interquartile range, the upper border of the box is the value demarcating the 3^rd^ and 4^th^ interquartile range, and the upper and lower whiskers are the samples highest and lowest values, respectively. The shaded box represents the known normal ranges for the TEG values. WB = whole blood (undiluted). *P<0.05.

The TEG k parameter is shown in Fig 2 for the volunteers, the trauma patients, and a PEG dose response series of diluted blood. The trend in all groups was a significant increase in the amplification time in both HES and PEG diluted whole blood compared to saline diluted blood. There were no differences in the whole blood and saline-diluted blood in the trauma group, for any parameter. While there was a significant difference between normal saline and both the 10% and 7.5% PEG dilutions within the healthy volunteer dose-response group, the elevated K time was returned to the normal range for both 7.5% and 5% solutions in a dose-dependent manner, simply by decreasing the concentration of PEG in the LVR solution.

### Clot propagation

**Fig 3:**
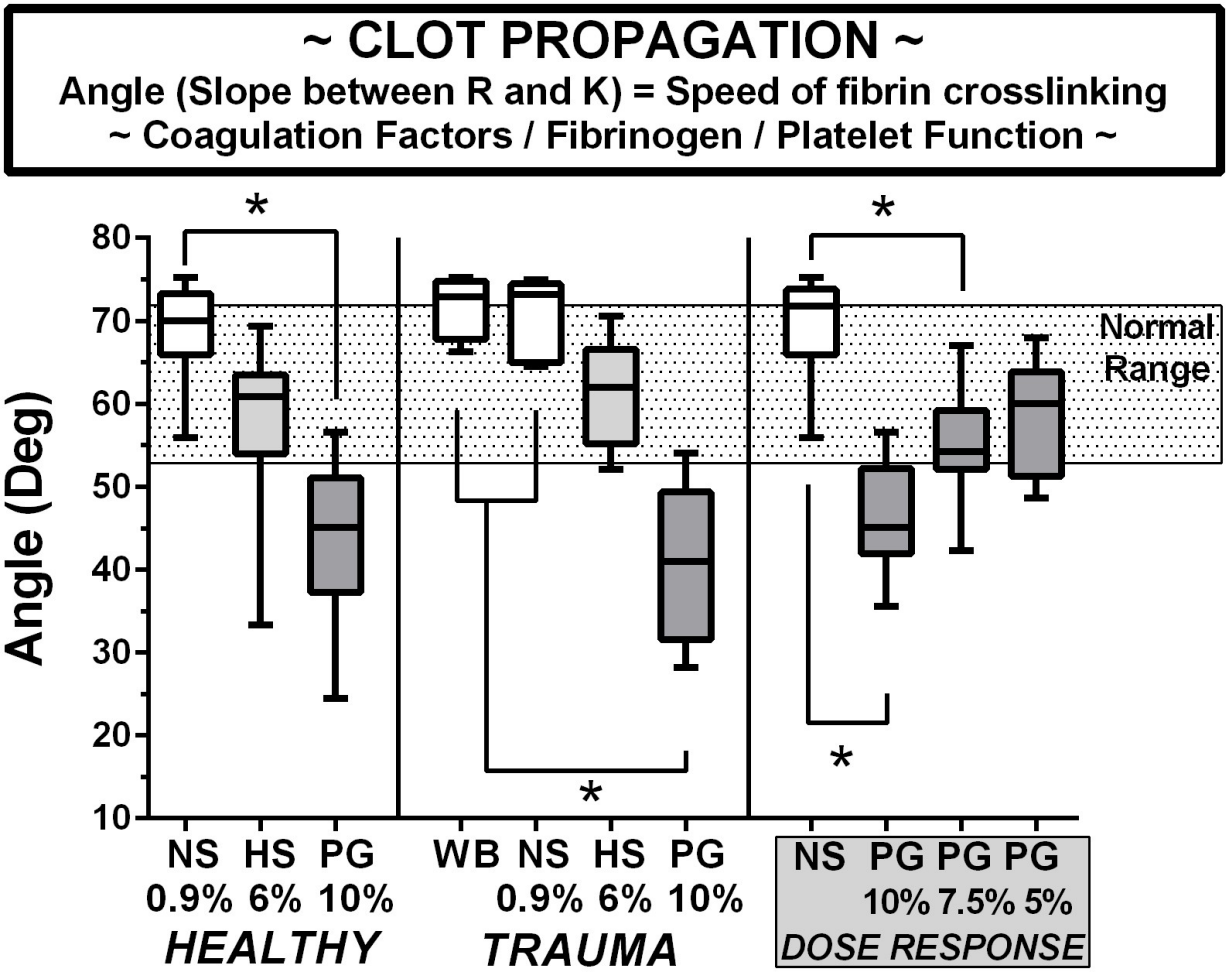
Clot propagation indexed by the Angle variable on TEG in blood from healthy volunteers, trauma patients, and volunteers in a PEG-20k dose-response series. All TEG outcomes represents 25 healthy volunteers, 9 trauma patients, and 9 volunteers for dose-response studies. Whole citrate preserved blood was immediately diluted 10% with a saline vehicle (NS), 6% solution of Hextend (HS), and Polyethylene Glycol-20k (PG) at concentrations of 10%, 7.5%, or 5% and assayed by full thromboelastography (TEG) within 2–3 hours of blood draw in a matched design with saline always serving as control to the resuscitative fluid. All values are expressed in a box and whiskers standard format where the bar in the box is the sample median value, the lower border of the box is the value demarcating the 1^st^ and second interquartile range, the upper border of the box is the value demarcating the 3^rd^ and 4^th^ interquartile range, and the upper and lower whiskers are the samples highest and lowest values, respectively. The shaded box represents the known normal ranges for the TEG values. WB = whole blood (undiluted). *P<0.05.

Fig 3 shows the data for the TEG angle parameter in volunteers, trauma patients, and healthy volunteer blood in the PEG dose-response study. The angle substantially decreases in both HES and PEG diluted blood compared to saline, with significant decreases in PEG groups. This effect is qualitatively and quantitatively the same in blood obtained from both volunteers and trauma patients. The significant decrease in the angle or propagation rate by 10% PEG-20k was normalized by reducing the concentration to 7.5% and 5% in the LVR solutions, seen in the dose-response section, similar to k.

### Maximum clot strength

**Fig 4:**
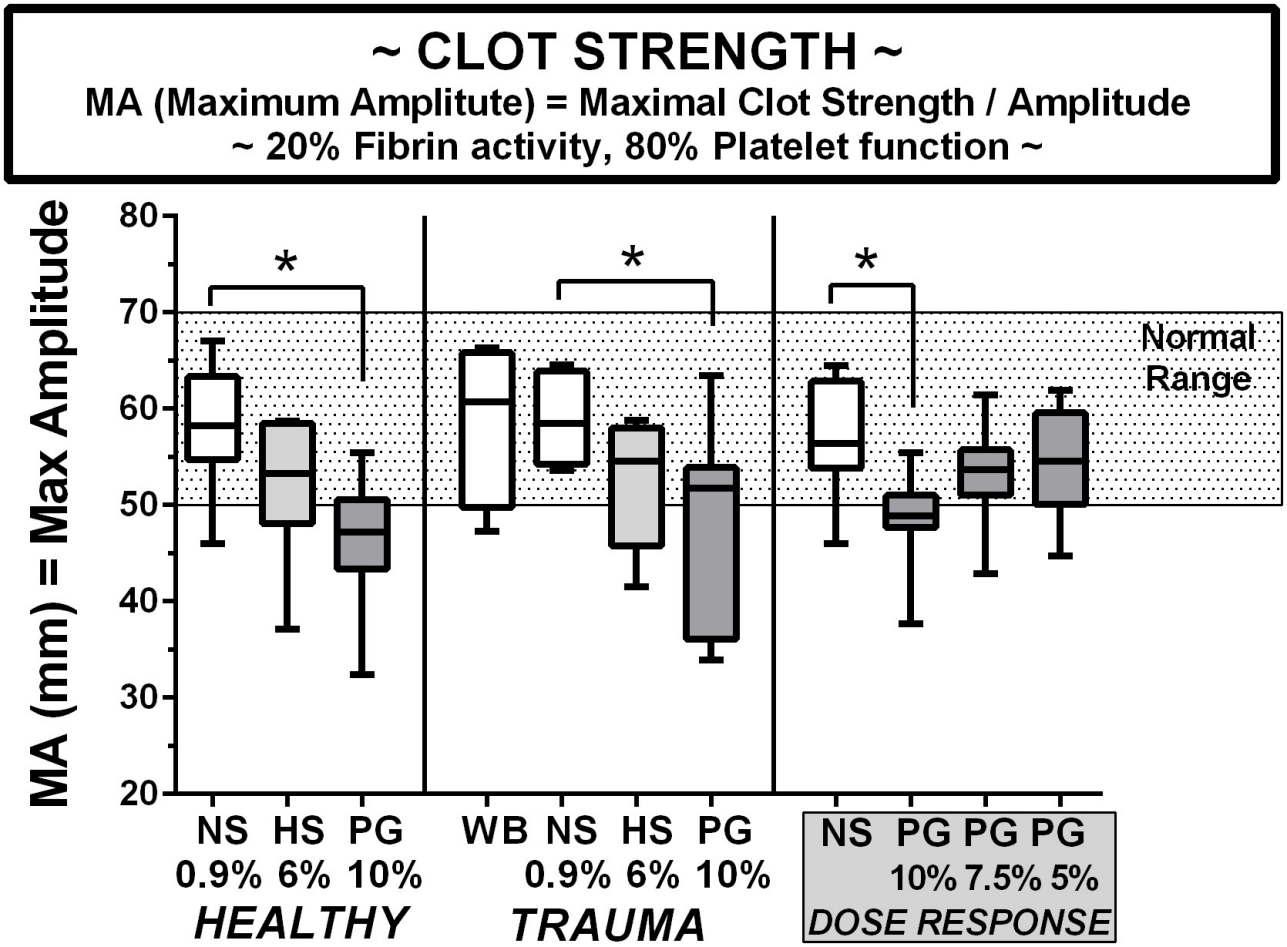
Clot strength and maximum size indexed by the MA on TEG in blood from healthy volunteers, trauma patients, and volunteers in a PEG-20k dose-response series. All TEG outcomes represents 25 healthy volunteers, 9 trauma patients, and 9 volunteers for dose-response studies. Whole citrate preserved blood was immediately diluted 10% with a saline vehicle (NS), 6% solution of Hextend (HS), and Polyethylene Glycol-20k (PG) at concentrations of 10%, 7.5%, or 5% and assayed by full thromboelastography (TEG) within 2–3 hours of blood draw in a matched design with saline always serving as control to the resuscitative fluid. All values are expressed in a box and whiskers standard format where the bar in the box is the sample median value, the lower border of the box is the value demarcating the 1^st^ and second interquartile range, the upper border of the box is the value demarcating the 3^rd^ and 4^th^ interquartile range, and the upper and lower whiskers are the samples highest and lowest values, respectively. The shaded box represents the known normal ranges for the TEG values. WB = whole blood (undiluted). *P<0.05.

The TEG MA parameter is shown in Fig 4 for blood from healthy volunteers, in blood from trauma patients, and volunteer blood in a PEG-20k dose-response series. The maximum strength or clot size is generally believed to represent a contribution by both platelets (80%) and fibrin (20%) under these conditions. While, the MA response is reduced in both HES and PEG diluted blood, significantly so in the 10% PEG-20k group relative to the saline dilutional controls, the effect is less severe than seen with either parameters k or angle, given the proximity of the means to the outer limits of normal range and absolute difference between means of the groups. The patterns again are almost identical in blood obtained from both healthy volunteers and trauma patients, as no significant differences exist between the groups. The significantly lower clot strength in blood diluted with 10% PEG-20k could again be dose-dependently reversed by progressively lowering the PEG concentration in the LVR solutions to 7.5% and 5%.

### Clot lysis

**Fig 5:**
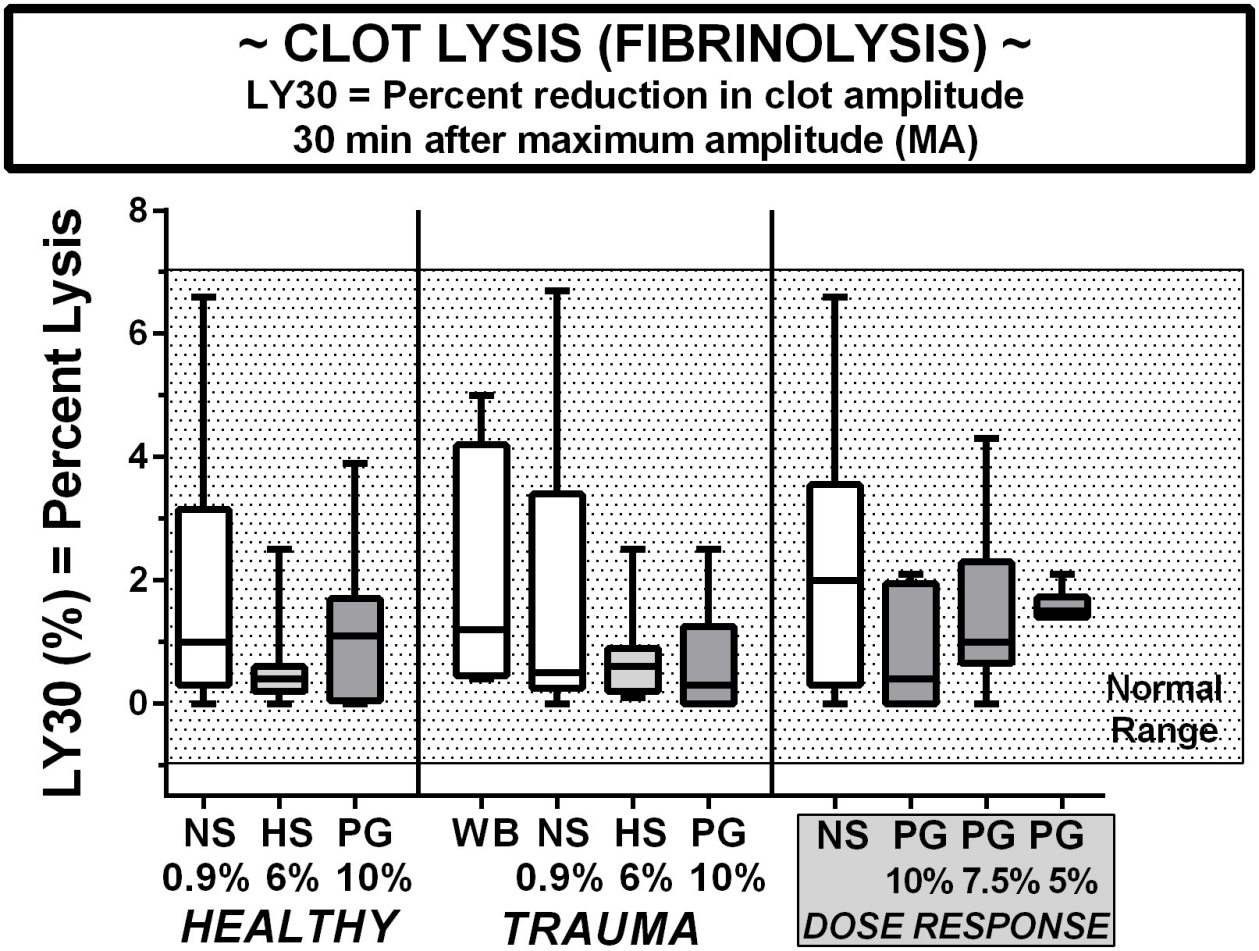
Clot lysis indexed by the LY30 on TEG in blood from healthy volunteers, trauma patients, and volunteers in a PEG-20k dose-response series. All TEG outcomes represents 25 healthy volunteers, 9 trauma patients, and 9 volunteers for dose-response studies. Whole citrate preserved blood was immediately diluted 10% with a saline vehicle (NS), 6% solution of Hextend (HS), and Polyethylene Glycol-20k (PG) at concentrations of 10%, 7.5%, or 5% and assayed by full thromboelastography (TEG) within 2–3 hours of blood draw in a matched design with saline always serving as control to the resuscitative fluid. All values are expressed in a box and whiskers standard format where the bar in the box is the sample median value, the lower border of the box is the value demarcating the 1^st^ and second interquartile range, the upper border of the box is the value demarcating the 3^rd^ and 4^th^ interquartile range, and the upper and lower whiskers are the samples highest and lowest values, respectively. The shaded box represents the known normal ranges for the TEG values. WB = whole blood (undiluted).

The TEG Ly30 data are provided in Fig 5. The rate of clot lysis was mostly less than 1–2% after 30 minutes and was not affected by the dilution with any LVR solution, including PEG-20k. The rate of fibrinolysis was also not different in the trauma patients compared to healthy volunteers. As opposed to the other TEG parameters, NS and WB groups had much larger ranges than the HS or PG counterparts. Additionally, there appears to be a nonsignificant but slightly notable trend of higher concentration PEG to dampen any fibrinolytic response measured under these ex-vivo settings.

### Coagulation index

**Fig 6:**
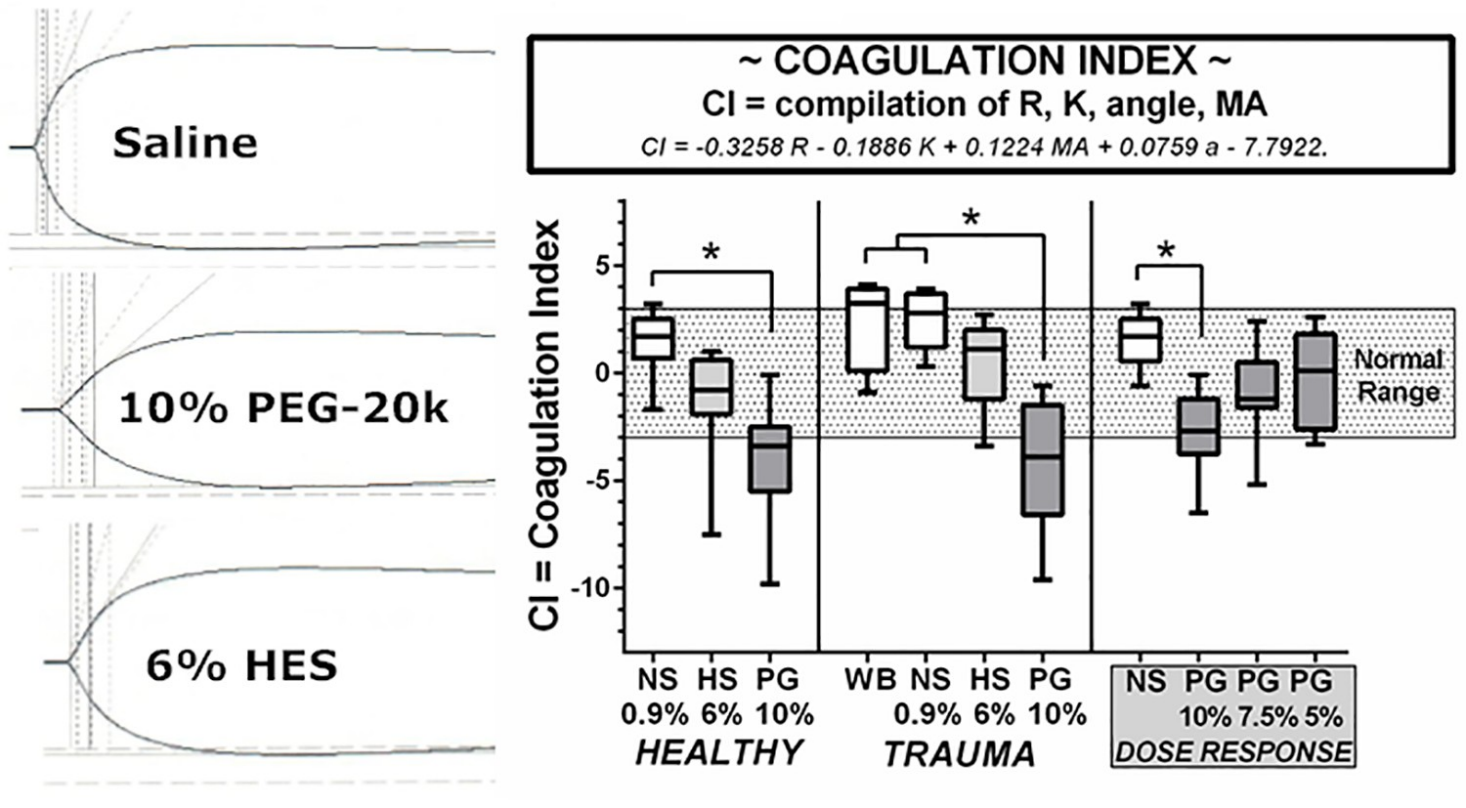
Coagulation Index as measured by the CI on TEG in blood from healthy volunteers, trauma patients, and volunteers in a PEG-20k dose-response series. The CI is a mathematical compilation of other TEG outcome variables (R, K, Angle, and MA). All TEG outcomes represents 25 healthy volunteers, 9 trauma patients, and 9 volunteers for dose-response studies. Whole citrate preserved blood was immediately diluted 10% with a saline vehicle (NS), 6% solution of Hextend (HS), and Polyethylene Glycol-20k (PG) at concentrations of 10%, 7.5%, or 5% and assayed by full thromboelastography (TEG) within 2–3 hours of blood draw in a matched design with saline always serving as control to the resuscitative fluid. All values are expressed in a box and whiskers standard format where the bar in the box is the sample median value, the lower border of the box is the value demarcating the 1^st^ and second interquartile range, the upper border of the box is the value demarcating the 3^rd^ and 4^th^ interquartile range, and the upper and lower whiskers are the samples highest and lowest values, respectively. The shaded box represents the known normal ranges for the TEG values. WB = whole blood (undiluted). Tracings on the left are representative TEG tracings obtained from all three LVR solutions used in this study (NS, PEG-20k, and HES). *P<0.05.

The coagulation index is shown in Fig 6 for the blood dilutions in the healthy volunteers, the trauma patients, and the volunteers in the PEG-20k dose-response study. The coagulation index (CI) is a mathematical compilation of other TEG parameters and is described by CI = -0.3258R-0.1886K+0.1224MA+0.0759α-7.7922. The normal range for CI is between 3.0 and -3.0. As shown in Fig 6, blood from either healthy volunteers or trauma patients had substantial reduction in the CI with HES and significant reduction with PEG-20k relative to saline control, similar to trends discussed in other parameters. However, there is NO statistical difference from lower limit of normal for any group. Of note, to the left of the CI panel are representative TEG tracings to indicate increasing level of hypocoagulability with narrowing tracing. The CI could be dose dependently reversed into the normal range by reductions in the concentration of the PEG-20k in the LVR solution.

### Resuscitation performance of 7.5% PEG-20k solutions

**Fig 7:**
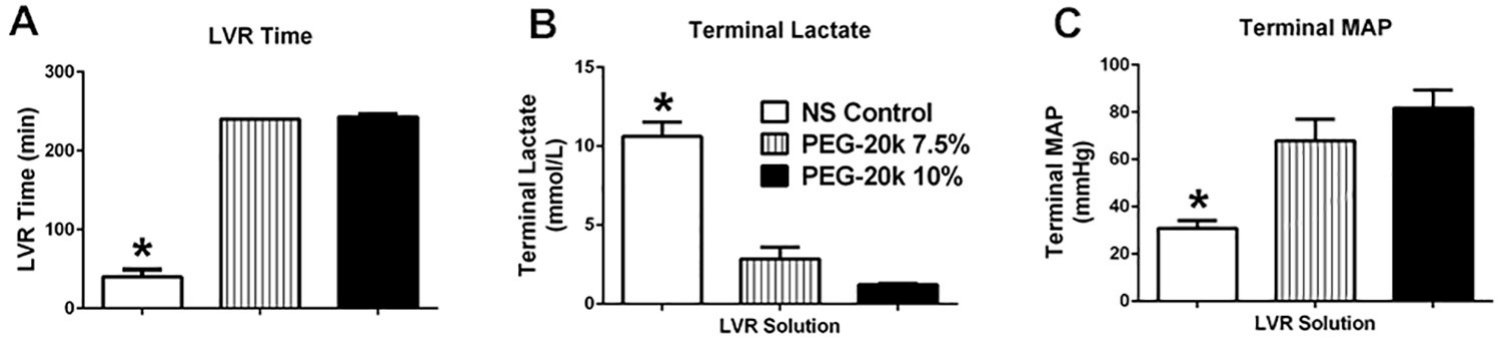
Acute resuscitation outcomes in a study in rats subjected to severe hemorrhagic shock and low volume resuscitation with NS (saline) controls, or 7.5% and 10% PEG-20k. All LVR solutions were given at a volume equal to 10% of the estimated blood volume of the animals. The low volume resuscitation times (LVR) were measured and shown in panel A, which is an index of tolerance to the low volume state (see Methods for details). The end or terminal lactate values are shown in panel B, which are the values at the end of the LVR period (or 240 minutes in the PEG groups), and the terminal mean arterial blood pressure measured at the end of the LVR period in panel C. Values are mean +/- standard deviation, n = 5 for NS and 10% PEG-20k and n = 4 for 7.5% PEG-20k. Baseline MAP before hemorrhage was 101 +/- 18 mmHg and baseline lactate before hemorrhage was 0.9 +/- 0.2 mM/L, *P<0.05 relative to both PEG groups.

Because slight reductions in the concentration of PEG-20k in LVR solutions (to 7.5% or 5%) caused a normalization of TEG parameters relative to those observed using 10% PEG, the effects of the reduced concentration on resuscitation outcomes was determined in our common rodent hemorrhagic shock model. Six Sprague Dawley rats were used for each group but one was excluded in the 7.5% PEG-20k group (total n = 14). Fig 7 shows that reducing the concentration of PEG-20k from 10% to 7.5% produces an equivalent resuscitation effect to the one observed with 10% PEG-20k. This is true when the quality of the resuscitation is described by the LVR time (panel A), the terminal lactate values (panel B), or the terminal mean arterial blood pressures (MAP, panel C). LVR time (i.e. tolerance to the low volume state) of either PEG-20k solution (10% or 7.5%) is approximately six times that of saline (40 ± 20.3 vs. 240 ± 6.7 minutes), although true LVR time is unknown as experiments were stopped at 240 minutes. End lactate of 7.5% PEG (2.9 ± 1.5 mmol/L) is approximately a quarter of saline (10.6 ± 2 mmol/L), while 10% PEG is approximately one eighth compared to saline (1.2 ± 0.13 mmol/L). MAPs are also approximately 2.5 times higher with PEG solutions (67.8 ± 7.2 and 81.6 ± 17.6 mmHg for PEG 7.5% and 10%, respectively), vs saline (30.9 ± 7.2 mmHg).

## Discussion

### Low volume resuscitation and polyethylene Glycol-20k

Low volume crystalloid resuscitation is used in early pre-hospital resuscitation of severely shocked patients in civilian and military settings for two important reasons. First because lower dilutional volume replacement has superior outcomes compared to the traditional large volume resuscitation strategies [13] and second, because low volume crystalloid is friendly to resource poor austere environments of distant field locations, especially in forward military theatres and geographically challenging regions.

Therefore, a new crystalloid low volume resuscitation solution has been developed and tested in pre-clinical hemorrhagic and trauma shock models and found to be highly effective in increasing the tolerance to the low volume state by significantly increasing microcirculatory oxygen transfer efficiency. These solutions are based on specifically sized polymers of polyethylene glycol solutes (PEG-20k) that work by osmotic and hydrophilic actions in the microcirculation. These forces non-energetically move isotonic fluid out of metabolically swollen cells into capillaries thereby reloading the exchange vessels and propelling convective oxygen transfer by decreasing the resistance to flow via their primary effects on cell and tissue swelling (tissue decompression). The result is rapid oxygen debt repayment, lactate clearance, and re-establishment of oxygen transfer under very low volume conditions.

This approach is ideal for pre-hospital use because metabolic and cardiovascular tolerance to trauma increases, which can safely lengthen evacuation and transport times and ensures better outcomes when definitive resuscitation occurs at a civilian or forward military hospital [8]. Since the active molecules in these new solutions are large polymers not unlike hydroxyethyl starch (HES) and because they produce significant water transfer and dilutional effects in blood compartments, the effects of these solutions on whole blood coagulation and platelet function are of possible concern and are as yet unknown. Therefore, the purpose of this study was to characterize the effects of LVR solutions containing PEG-20k on whole blood coagulation and platelet function using TEG analysis (this report) and on more detailed mechanisms of coagulation and platelet function using more specific testing in a companion paper to this one.

### Extrapolating ex-vivo results to in-vivo responses

In these experiments using ex-vivo diluted whole blood, 10% PEG-20k produces a clinically recognizable coagulopathy (i.e. requiring blood product transfusion per ACS TQIP Massive Transfusion in Trauma Best Practice Guidelines) that is statistically different from normal saline dilutional control at a 10% volume dilution, but *not* from 6% HES colloidal controls in healthy volunteers or trauma patients. Additionally, it appears the effects at 10% PEG may be attenuated with lower concentrations.

The dilution factor of 10% was chosen since this is the upper limit of low volume resuscitation ranges that may be used in the field, corresponding to an approximate volume of 500 ml in an adult (with a blood volume of 5 liters). Since these studies are diluted ex-vivo, we assume the coagulation effects observed are true for patients when they are diluted at a similar 10% volume. However, in trauma patients in the field requiring low volume resuscitation, there is no way of accurately estimating if a theoretical 10% LVR dilution with crystalloid actually represents 10% or something larger or smaller. Understanding this is essential in order to extrapolate the coagulation results from this ex-vivo study that used an exact 10% dilution with the LVR solutions being tested.

The *forces favoring a greater dilution in the trauma patient* over the 10% estimated theoretical value (resulting in a lower % PEG-20k concentration) include dilution in the vascular space from subsequent movements of isotonic water from the intracellular and interstitial spaces into the capillary space, which is where coagulation and platelet function occurs. This is probably a significant dilution and can represent a doubling of the intravascular isotonic water volume that cuts hemoglobin and albumin marker concentrations in half [8, 10]. Essentially, an auto-infusion with the body’s own isotonic fluid being driven by the PEG low volume resuscitation.

An additional factor that would favor a dilution of the PEG exceeding the theoretical calculated 10% after administration to trauma patients include the capillary reflection coefficient of the solute (PEG-20k). The osmotic reflection coefficient σ_d_ is a measure of the % partitioning of a large solute molecule (like PEG-20k) between the capillary and the extracapillary space. A molecule that has a reflection coefficient of 1.0 demonstrates 100% reflection by pores in the capillary so 100% stays in the capillary available for interactions with coagulation factors and platelet interactions. A coefficient of 0 indicates no reflection and the solute is equilibrated equally between the capillary and interstitial spaces, or, 50% of the material and the osmotic effect is lost. The actual σ_d_ of PEG-20k is 0.5 in most capillary beds [2, 10], which means that 33% of the material administered into the vascular space (10% theoretically) quickly equilibrates outside of the capillary into the interstitial space. In fact, this intermediate reflection coefficient, which is rare, was a sought-after molecular attribute for choosing an ideal impermeant solute to construct an LVR solution with maximum water transfer properties. This means that, all things being equal, administration of a 10% PEG-20k solution will result in a 6.6% solution after the solute molecules equilibrate across the capillary membrane, based on the properties of the capillary as defined by PEG-20k’s unique reflection coefficient. This property, along with the large pull of water into the capillary space from the osmotic and hydrophilic forces of the PEG-20k, tends to dilute out the PEG-20k concentration in the blood and reduce the effects of the PEG polymers on interference with coagulation.

The *factors that tend to increase the concentration* of PEG-20k from the theoretical 10% dilution after administration to trauma patients is the large hemorrhage volumes that are not taken into account when the theoretical blood volume is calculated. If 25% of the blood volume is lost in a trauma patient to hemorrhage, then administration of a 10% solution at an estimated 10% blood volume dilution will result in an underestimation of the blood volume and a more concentrated final PEG-20k concentration in the vascular space (to about 12.5%). This may tend to exacerbate any dose-dependent PEG-20k effects on the coagulation and platelet system. The final dilution of a 10% PEG-20k solution given at a theoretical 10% dilution in a trauma patient will be an algebraic sum of all of these forces acting together. Our preliminary modeling of a patient with a 40% hemorrhage volume suggest a dilution greater than 10%, which would lessen the coagulation side effects that had been documented here in ex-vivo whole patient blood at an exact 10% dilution. This has been validated with some preliminary animal hemorrhage studies using labeled PEG-20k. Finally, we know that we can move the PEG-20k dilution down to 7.5% and be both effective in shock resuscitation (rodent study) and neutral with respect to changing TEG parameters of coagulation and platelet function.

### Proposed coagulopathy mechanisms

The mechanism of the coagulopathic effects of PEG-20k LVR solutions on clotting blood cannot be determined from the TEG data because the tests are descriptive. However, certain mechanistic effects can be inferred from the individual changes in the TEG outcome variables. The TEG responses in both healthy volunteer and trauma patient blood diluted 10% with PEG-20k solutions (at 10% concentration) suggests mainly an interference with direct platelet function (because MA is reduced) and possibly indirect effects of the platelet contribution to coagulation from thrombin generation (because k and angle are affected). However, direct platelet aggregation tests were not performed in this study so a detailed analysis of this contribution to our TEG results is not possible. The R value was slightly elevated suggesting a mild enzymatic initiation defect too. Other possible effects on coagulation reactions cannot be excluded from the TEG data alone.

### Proposed clinical utility

The effects of PEG-20k on coagulation and platelet function, as assessed by TEG, were the same in blood from health volunteers and trauma patients, despite widely disparate presumed baseline physiology during a shock state. This is good to know since our intent is to understand how these solutions influence systems in the trauma patient. While this study is limited by the ex-vivo format, that is, lacking the endothelial aspect of coagulation in real time, or the physiologic changes of shock states, the matched controls in an ex-vivo setting allows for controlled evaluation of any baseline effects of PEG opposed to military colloid (HES) or civilian crystalloid (NS) controls.

Our selection criteria for trauma patients was strict in that we wanted severely injured patients with an Injury Severity Score (ISS) over 24, a lactate on arrival of ≥ 4.6 mM, or hypotension characterized by a systolic blood pressure <95 mmHg. Another goal was to measure patients as soon as they entered the trauma system because they would have a greater chance of not being transfused with blood or given significant volumes of crystalloids that would further complicate an already chaotic system. We selected a small but diverse population including multiple injury mechanisms and outcomes, with ISS averaging 30 (range 9–48) and SBPs averaging 91 mm Hg (range 50–124). Lactate was not as impressive, but did average 3.3 mM with range 1–5 mM. Given the severity of illness, it was surprising that we didn’t see evidence of a trauma induced coagulopathy (TIC) in our trauma patient population, especially a temporary hyper-coagulative state. We also did not follow these patients in time to document the development of a later TIC or hypocoagulative state. In any case, the effects of PEG-20k LVR solutions behaved almost identically in patients as it did in healthy volunteers.

### Conclusions

This study compared various concentrations of PEG-20k on coagulation and platelet function using TEG compared to a dilutional saline control and a clinical (military medicine) control using a 6% solution of hydroxyethyl starch (Hextend). While both Hextend and PEG-20k solutions produced measureable and significant effects on TEG outcomes, the PEG effects were not significantly different from the Hextend effects even though the absolute changes appeared more pronounced in the 10% PEG-20k groups. The commonality between PEG-20k and HES are that they both are polymers and they both have hypocoagulative effects on whole blood TEG testing. However, these polymers are chemically different and the similarity or differences in their mechanisms of action on the coagulation and platelet activation system should be speculated with caution until more definitive mechanistic testing is performed. At this point, it appears that the qualitative effects on TEG for both polymers are very similar when comparing 6% HES with 10% PEG-20k.

In conclusion, this study clearly shows that LVR solutions used for the resuscitation of patients in severe hypovolemic shock has statistically significant but minor effects on whole blood coagulation and platelet function as determined by TEG in an ex-vivo test system. The effects are not due to volume dilution and are similar to those seen with 6% HES. The PEG-20k effects are dose dependent and are essentially abrogated and reversed by reducing the PEG-20k concentrations from 10% to 7.5%. The exact mechanisms of the polymer effects on TEG are not known, but the TEG analysis suggests that platelet function or fibrinogen conversion may be involved. The clinical effects will need to be verified in an in-vivo model.

## Supporting information

S1 File Original data files and statistical analysis for all figures presented in Prism 6.0 format. (PZF)

## Abbreviations

0.9% NaCl: NS, Normal saline
HES: Hydroxyethyl Starch
HS: HEXTEND
IRB: Institutional Review Board
ISS: Injury severity score
JTTS CPG: Joint Theater Trauma Systems Clinical Practice Guidelines
LR: Lactated Ringer’s
LVR: Low volume resuscitation
PEG-20k: Polyethylene glycol (PEG) 20,000 mw
TEG: Thromboelastography
TIC: Trauma induced coagulopathy
TICS: Trauma-induced capillary leak syndrome
VCU: Virginia Commonwealth University
WB: Whole Blood, undiluted
